# Supplementary material for: Ad26 vector-based COVID-19 vaccine encoding a prefusion-stabilized SARS-CoV-2 Spike immunogen induces potent humoral and cellular immune responses
Source: NPJ Vaccines. 2020 Sep 28;5:91. doi: 10.1038/s41541-020-00243-x (PMC7522255; doi:10.1038/s41541-020-00243-x)
Supplement: Supplementary file 1 — Supplementary Information [file 41541_2020_243_MOESM1_ESM.pdf]

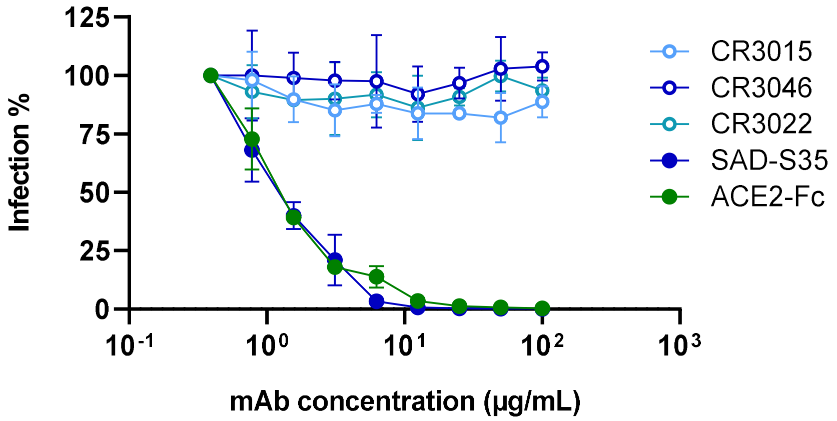

Supplementary Figure 1. Antibody-mediated neutralization of MLV particles pseudotyped with S protein of SARS-CoV-2. Pseudotyped MLV particles pre-incubated with monoclonal antibodies or soluble receptor ACE2-Fc in a concentration range were used to transduce Vero E6 cells. The luminescence signal of the luciferase activity in transduced Vero E6 cells was measured 40 h post transduction to calculate the infection (%) of the Vero E6 cells relative to non-treated MLV particles.

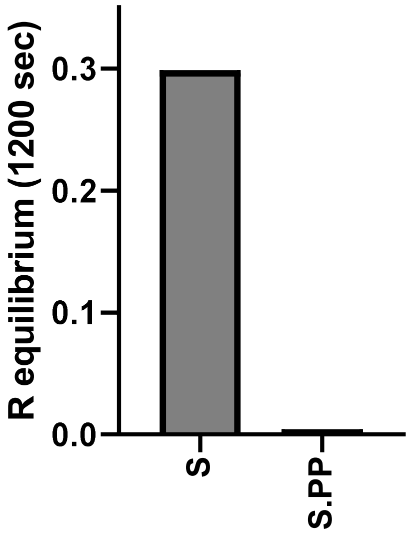

Supplementary Figure 2. Shedding of SARS-CoV-2 S1. Biolayer interferometry to determine the binding of ACE2 to Expi293F cell culture supernatants expressing S and S.PP three days after transfection.

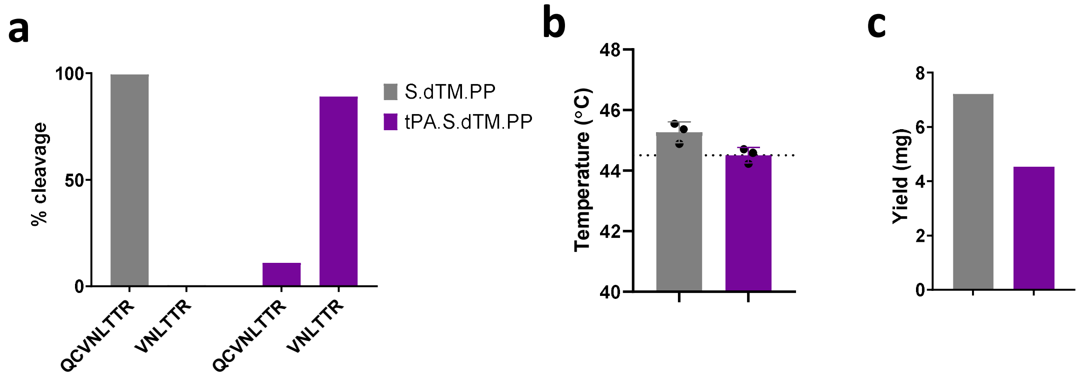

Supplementary Figure 3. Soluble protein characterization. a) Percentage of SP cleavage at cleavage site 1 (CS1) resulting in peptide QCVNLTTR and at cleavage site 2 (CS2) resulting in peptide VNLTTR (see Fig 3A) as determined with mass-spectrometry. **b**) Melting temperature determined with DSF (n=3). The dotted horizontal line indicates the height of the Tm_{50} of tPAs.S.dTM.PP. Data are represented as mean ±SD and with the separate three data points. **C**) Protein yield per 900 ml culture for secreted S proteins.

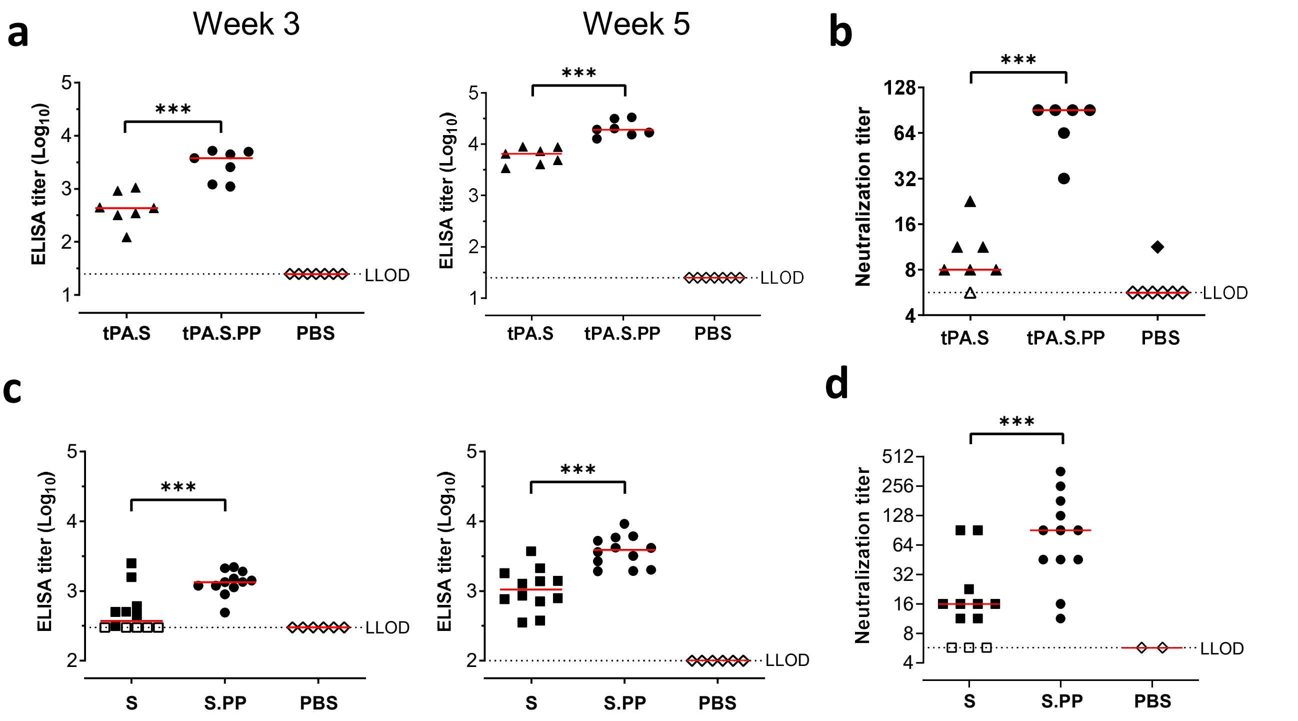

Supplementary Fig 4. S protein-specific antibody binding titers and SARS-CoV-2 NAbs induced by DNA immunization. Naïve mice (BALB/c) were immunized twice, 3 weeks apart (Week 0 and Week 3), with 100 µg of DNA constructs. Serum samples were taken prior to the second immunization (Week 3), and 2 weeks after the second immunization (Week 5). Spike protein-specific binding and SARS-CoV-2 NAb titers were determined. In two separate experiments, mice were immunized with either **a** + **b**) tPA.S (N=7), tPA.S.PP (N=7), or PBS (N=7), or **c** + **d**) were immunized with either S (N=12), S.PP (N=12), or with PBS (N=6). Spike protein binding antibody titers were measured by ELISA. SARS-CoV-2 NAb titers were measured by wt VNA determining inhibition of the cytopathic effect (CPE) of virus isolate Leiden1 (L-0001) on Vero E6 cells. Mice immunized with PBS were taken along as two separate pools in fig S2**d**. The median response per group is indicated with a horizontal line. The dotted lines indicate LLODs. Animals with a response at or below the LLOD were put on LLOD and are shown as open symbols. Statistically significant differences as determined by z-test from Tobit ANOVA are indicated by asterisks ; **: p<0.01, ***p<0.001.

a CD8$^+$ T cells

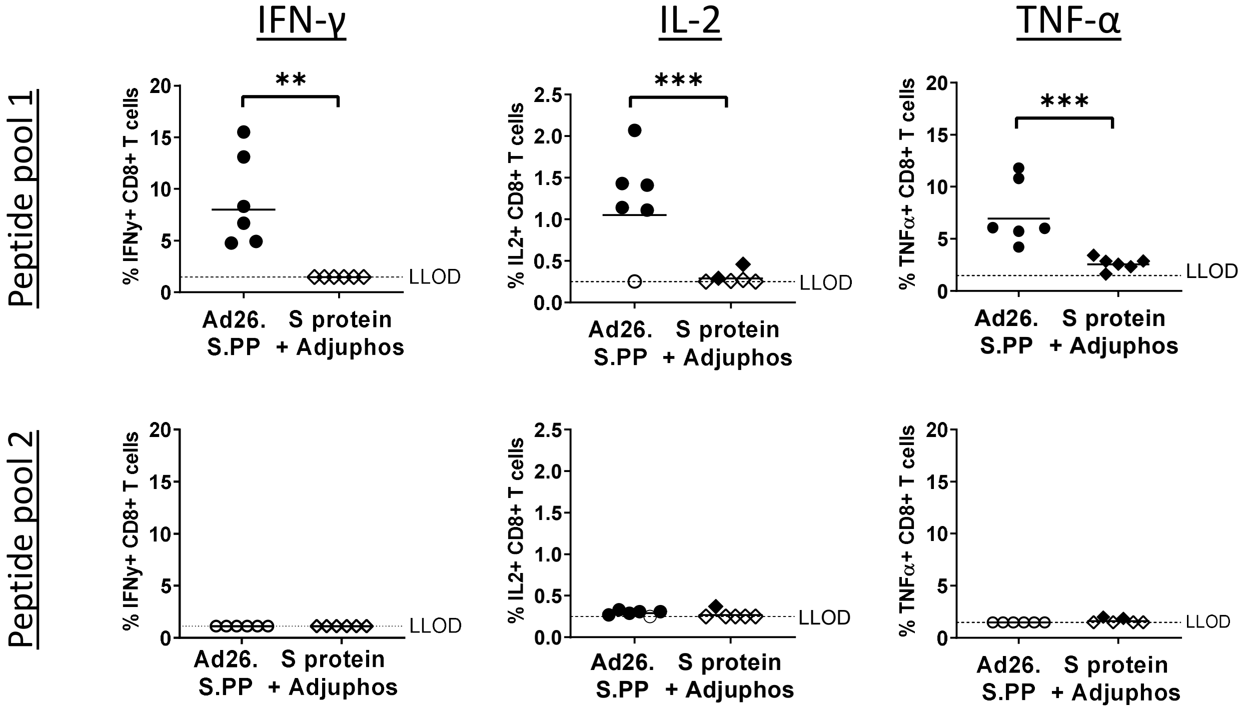

b CD4$^+$ T cells

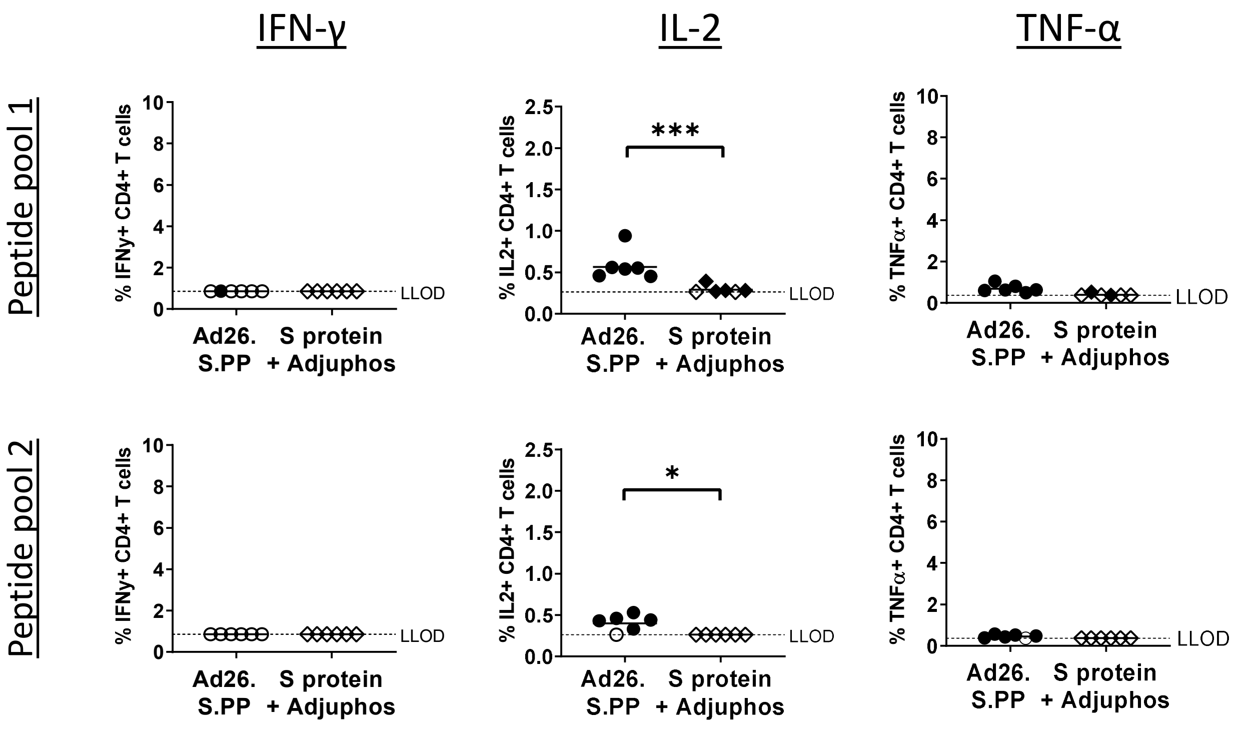

Supplementary Fig 5. Th1 associated cytokines induced by Ad26.S.PP compared with Adjuphos adjuvanted protein. Naïve mice (BALB/c, N=6 per group) were immunized with either 10^{10} vp of Ad26.S.PP or 50 mcg of Spike protein adjuvanted with 100 µg Adjuphos (Adju-Phos®). Two weeks after immunization splenocytes were analyzed for intracellular cytokine expression of IFN-γ, IL-2 and TNF-α after stimulation with SARS-CoV-2 Spike protein peptides. **a**) CD8 positive T cells and **b**) CD4 positive T cells were stimulated with either S protein peptide pool 1 or peptide pool 2.

The median response per group is indicated with a horizontal line. The dotted lines indicate LLODs. Animals with a response at or below the LLOD were put on LLOD and are shown as open symbols. Statistically significant differences as determined by Mann-Whitney U test, or a z-test from Tobit ANOVA when the majority of samples were above LLOD, indicated by asterisks; *:p<0.05, **: p<0.01, ***p<0.001.

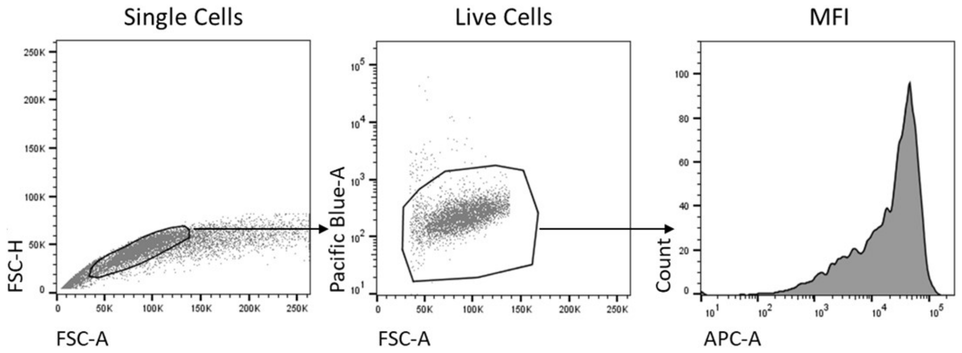

Supplementary Figure 6. Gating strategy in flow cytometry (see Fig. 2a). Gating on MRC-5 single, live cells, to obtain median fluorescence intensity in APC channel. Example shown is S309 binding to tPA.S.PP.

ID number	name	sequence
2972	CAG4 rv	CCACGGGACGGGCTC
4146	COR Fw1	AAGATCTACAGCAAGCACAC
4147	COR Fw2	AACAGCAACAACCTGGACTC
4148	COR Fw3	GTGCCAGCTACCAGACACAG
4149	COR Fw4	TCCCCTTTGCTATGCAGATG
4150	COR Fw5	CAGCTTCAAAGAGGAACTGG
4151	COR Rv1	AACAGGTCCTGGGTAGAGTG
4152	COR Rv2	ATCTGGTGGCATTGAACACC
4153	COR Rv3	TCTTGTTGCTCTCTGTCAGC
4154	COR Rv4	GTCTTGTAGATCTGCTTCAC
4155	COR Rv5	TTGGCAGAGGCTCTAATCTC
4170	WH-CoV_TGseq_1	GCGCGAAAACTGAATGAGGAA
4171	WH-CoV_TGseq_2	TGTTCCCATAGTAACGCCAAT
4172	WH-CoV_TGseq_3	GTGTACGGTGGGAGGTCTATA
4174	WH-CoV_TGseq_5	TCACTGCATTCTAGTTGTGGTT
4175	WH-CoV_TGseq_6	ATCATCGCCGAGGAGAAACT
4176	WH-CoV_TGseq_7	GGAAAGTCCCTATTGGCGTTAC
4177	WH-CoV_TGseq_8	CCGCATCACCATGGTAATAGC
4178	WH-CoV_TGseq_9	TATAGACCTCCCACCGTACAC
4179	WH-CoV_TGseq_10	CGGGCAGCTTGTAGTTGTAAT
4180	WH-CoV_TGseq_11	ACCACAACTAGAATGCAGTGA
4181	WH-CoV_TGseq_12	AGTTTCTCCTCGGCGATGAT
4182	WH-CoV_TGseq_13	AACATCGCCAACCTCAAGATC
4183	WH-CoV_TGseq_14	GGTCATCAAAGTGTGCGAGTT
4184	WH-CoV_TGseq_15	CTACCTGCAGCCTAGAACCTT
4185	WH-CoV_TGseq_16	CAATTACCTGTACCGGCTGTT
4186	WH-CoV_TGseq_17	GACGTGAACTGTACCGAAGTG
4187	WH-CoV_TGseq_18	ACAGGACAAGAACACCCAAGA
4188	WH-CoV_TGseq_19	TCGGCAAGATCCAGGACAG
4189	WH-CoV_TGseq_20	CAGATCATCACCACCGACAAC
4190	WH-CoV_TGseq_21	AGCCTCTGATGATGTTGGACT
4191	WH-CoV_TGseq_22	AAGGTTCTAGGCTGCAGGTAG
4192	WH-CoV_TGseq_23	GGGCAGCTTGTAGTTGTAGTC
4193	WH-CoV_TGseq_24	CTTCGGTACAGTTCACGTCCT
4194	WH-CoV_TGseq_25	CTCTTGGGTGTTCTTGTCCTG
4195	WH-CoV_TGseq_26	GTTGAACTGGTTGGCGATCA
4196	WH-CoV_TGseq_27	GTTGTCGGTGGTGATGATCTG
4197	WH-CoV_TGseq_28	AACAGCCGGTACAGGTAATTG
4198	WH-CoV_TGseq_29	GCTGGGCATGTAGCTCGA
4199	WH-CoV_TGseq_30	AGCGACGAGTTCCCCACG

Supplementary Table 1. Primers used for PCR of Ad26 vaccine vectors to confirm the identity and integrity of the SARS-COV-2 Spike genes.

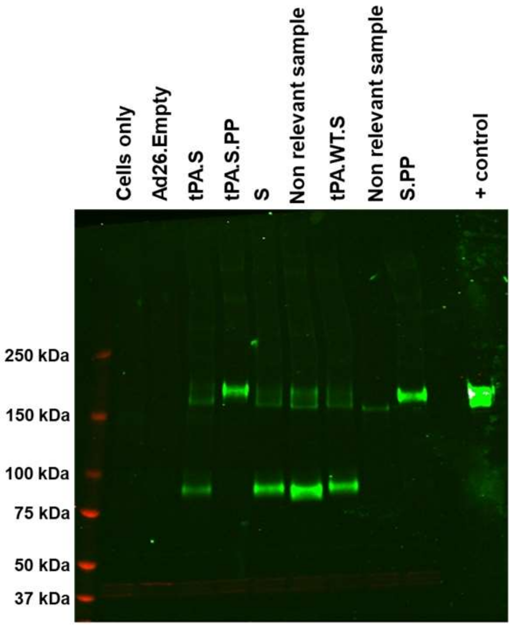

Supplementary Fig 7. Western blot analysis for expression from Ad26 vaccine vectors encoding tPA.S, tPA.S.PP, S, tPA.WT.S, and S.PP in MRC-5 cell lysates under non-reduced conditions using a human monoclonal antibody (CR3046). Ad26.Empty was included as negative control. SARS-CoV-2 S protein was included as positive control.

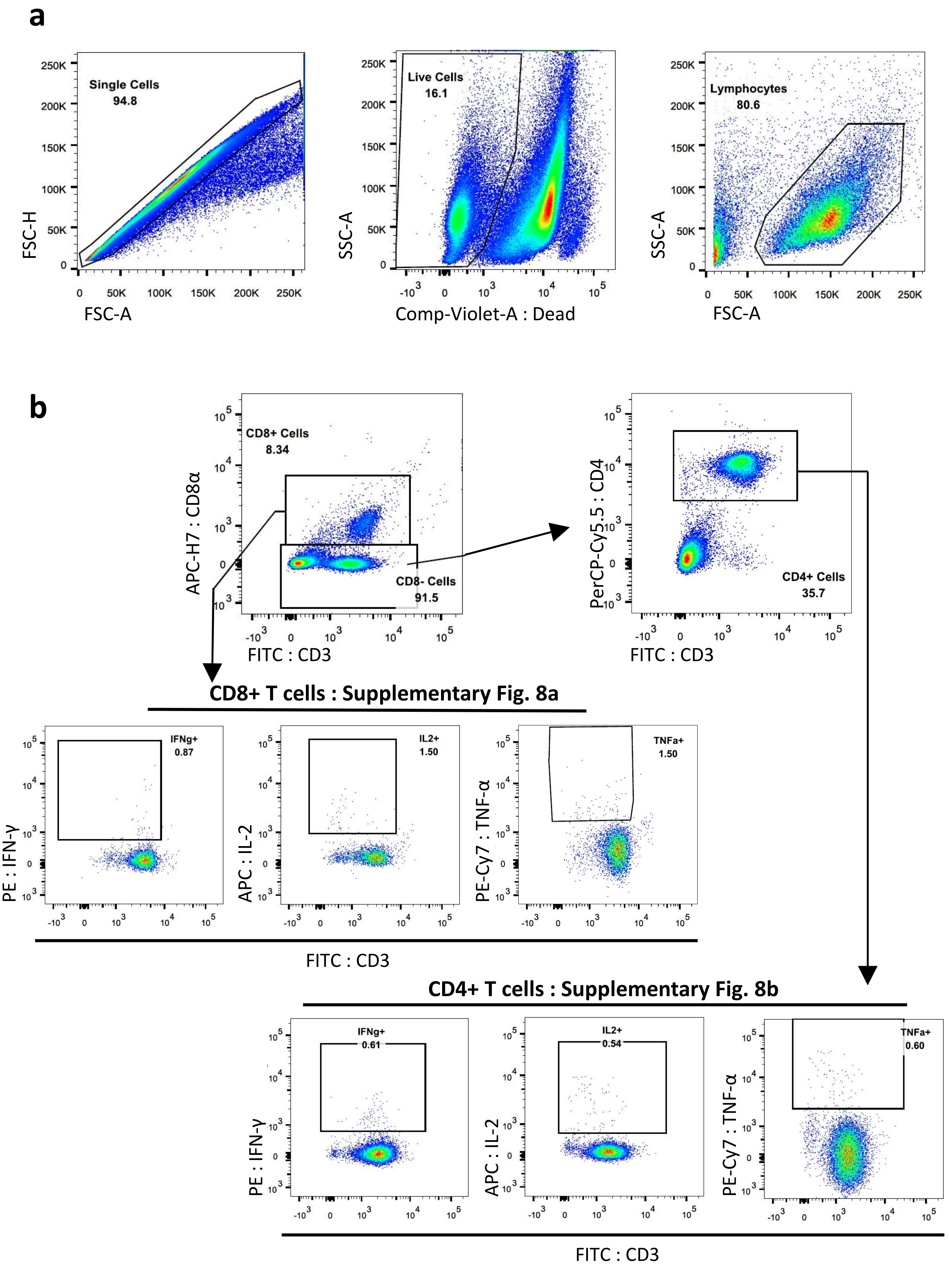
a
Single Cells
94.8
FSC-H
FSC-A
Live Cells
16.1
SSC-A
Comp-Violet-A : Dead
Lymphocytes
80.6
SSC-A
FSC-A
b
CD8+ Cells
8.34
CD8- Cells
91.5
APC-H7 : CD8α
FITC : CD3
CD4+ Cells
35.7
PerCP-Cy5.5 : CD4
FITC : CD3
CD8+ T cells : Supplementary Fig. 8a
IFNg+
0.87
PE : IFN-γ
IL2+
1.50
APC : IL-2
TNFa+
1.50
PE-Cy7 : TNF-α
FITC : CD3
CD4+ T cells : Supplementary Fig. 8b
IFNg+
0.61
PE : IFN-γ
IL2+
0.54
APC : IL-2
TNFa+
0.60
PE-Cy7 : TNF-α
FITC : CD3

Supplementary Fig 8. Gating strategy used for ICS assay. Spleens were harvested and splenocytes were analyzed for intracellular cytokine expression of IFN-γ, IL-2 and TNF-α after stimulation with SARS-CoV-2 Spike protein peptide pools. **a)** Single cells were selected by the FSC-A to FSC-H ratio, after which stained dead cells were excluded and lymphocytes selected. **b)** Subsequently, CD8+ T cells were selected by a positive CD8 staining, while CD4+ T cells were selected by a negative CD8 staining followed by a positive CD4 staining. Percentage of cytokine producing cells were measured by IFN-γ, IL-2 and TNF-α markers.
