# Supplementary material for: Ad26 vector-based COVID-19 vaccine encoding a prefusion-stabilized SARS-CoV-2 Spike immunogen induces potent humoral and cellular immune responses
Source: NPJ Vaccines. 2020 Sep 28;5:91. doi: 10.1038/s41541-020-00243-x (PMC7522255; doi:10.1038/s41541-020-00243-x)
Supplement: Supplementary file 2 — Reporting Summary [file 41541_2020_243_MOESM2_ESM.pdf]

nature research

Corresponding author(s): *Double-blind peer review submissions: write DBPR and your manuscript number here instead of author names.*

Last updated by author(s): Aug 27, 2020

Reporting Summary

Nature Research wishes to improve the reproducibility of the work that we publish. This form provides structure for consistency and transparency in reporting. For further information on Nature Research policies, see our Editorial Policies and the Editorial Policy Checklist.

Statistics

For all statistical analyses, confirm that the following items are present in the figure legend, table legend, main text, or Methods section.

n/a	Confirmed	
☐	☒	The exact sample size (*n*) for each experimental group/condition, given as a discrete number and unit of measurement
☐	☒	A statement on whether measurements were taken from distinct samples or whether the same sample was measured repeatedly
☐	☒	The statistical test(s) used AND whether they are one- or two-sided<br>*Only common tests should be described solely by name; describe more complex techniques in the Methods section.*
☐	☒	A description of all covariates tested
☐	☒	A description of any assumptions or corrections, such as tests of normality and adjustment for multiple comparisons
☐	☒	A full description of the statistical parameters including central tendency (e.g. means) or other basic estimates (e.g. regression coefficient) AND variation (e.g. standard deviation) or associated estimates of uncertainty (e.g. confidence intervals)
☐	☒	For null hypothesis testing, the test statistic (e.g. *F*, *t*, *r*) with confidence intervals, effect sizes, degrees of freedom and *P* value noted<br>*Give P values as exact values whenever suitable.*
☒	☐	For Bayesian analysis, information on the choice of priors and Markov chain Monte Carlo settings
☒	☐	For hierarchical and complex designs, identification of the appropriate level for tests and full reporting of outcomes
☒	☐	Estimates of effect sizes (e.g. Cohen's *d*, Pearson's *r*), indicating how they were calculated

Our web collection on statistics for biologists contains articles on many of the points above.

Software and code

Policy information about availability of computer code

Data collection	FACS and ICS data were collected with FlowJoTM Software version 10.06.1 (Becton, Dickinson and Company).
Data analysis	Statistical analyses were performed using SAS version 9.4 (SAS Institute Inc. Cary, NC, US) and R version 3.6.1 (2019-07-05). FACS and ICS analysis were performed with FlowJoTM Software version 10.06.1(Becton, Dickinson and Company) for analysis of flow cytometry.

For manuscripts utilizing custom algorithms or software that are central to the research but not yet described in published literature, software must be made available to editors and reviewers. We strongly encourage code deposition in a community repository (e.g. GitHub). See the Nature Research guidelines for submitting code & software for further information.

Data

Policy information about availability of data

All manuscripts must include a data availability statement. This statement should provide the following information, where applicable:

- Accession codes, unique identifiers, or web links for publicly available datasets
- A list of figures that have associated raw data
- A description of any restrictions on data availability

All data that support the findings of this study are available from the corresponding author upon reasonable request.

Field-specific reporting

Please select the one below that is the best fit for your research. If you are not sure, read the appropriate sections before making your selection.

☒ Life sciences ☐ Behavioural & social sciences ☐ Ecological, evolutionary & environmental sciences

For a reference copy of the document with all sections, see nature.com/documents/nr-reporting-summary-flat.pdf

Life sciences study design

All studies must disclose on these points even when the disclosure is negative.

Sample size	Each study had a predefined objective, and based on historical data obtained in-house, sample sizes were calculated by statisticians to detect predetermined effect sizes with 80% power.
Data exclusions	No data was excluded during analysis
Replication	All experiments included either control groups or control time-points validating the experiments. All experiments met the pre-set gatekeepers for the validity of the experiment. Experiments were not replicated due to ethical reasons of animal experimentation.
Randomization	Mice were randomly assigned to groups
Blinding	Investigators were blinded to group allocation during in vivo and in vitro data collection. Blinding was removed during data analysis after the experiment finished and results had been quality controlled

Reporting for specific materials, systems and methods

We require information from authors about some types of materials, experimental systems and methods used in many studies. Here, indicate whether each material, system or method listed is relevant to your study. If you are not sure if a list item applies to your research, read the appropriate section before selecting a response.

Materials & experimental systems

n/a	Involved in the study	
☐	☒	Antibodies
☐	☒	Eukaryotic cell lines
☒	☐	Palaeontology and archaeology
☐	☒	Animals and other organisms
☒	☐	Human research participants
☒	☐	Clinical data
☒	☐	Dual use research of concern

Methods

n/a	Involved in the study	
☒	☐	ChIP-seq
☐	☒	Flow cytometry
☒	☐	MRI-based neuroimaging

Antibodies

Antibodies used	SAD-S35 (ACROBiosystems)(cat# SAD-S35-100ug). ACE2-Fc, CR3022, CR3015, CR3046 and S309 were made in-house. For ICS the following antibodies were used: The following antibodies were used in the ICS assay: Rat-anti-Mouse CD49d (BD; cat #553153), Hamster-anti-Mouse CD28 (BD; cat #553294), anti-CD3e FITC (BD, cat#553062), anti-CD4 PerCP-Cy5.5 (BD, cat#550954), anti-CD8α APC-H7 (BD, cat#557654), anti-IFN-γ PE (BD, cat#554412), anti-TNF-α PE-Cy7 (557644) and anti-IL-2 APC (BD, cat#554429).
Validation	SAD-S35 and S309 are RBD-directed SARS-CoV-2 neutralizing Abs. SAD-S35 (Biosystems) antibody recognizes the SARS-CoV-2 Spike Protein RBD domain and inhibits the interaction between SARS-CoV-2 RBD and ACE2 with an IC50 of 1.47 µg/mL using SARS-CoV-2 Inhibitor Screening Kit (Cat. No. EP-105). CR3022, CR3015 and CR3046 bind SARS-CoV-2 S protein, but are non-neutralizing. CR3022 was made and characterized in-house.<br>The following antibodies were used in the ICS assay: co-stimulatory antibodies Rat-anti-Mouse CD49d (1:500, BD; cat #553153) and Hamster-anti-Mouse CD28 (1:500, BD; cat #553294). For identification of cell subsets anti-CD3e FITC (BD, cat#553062), anti-CD4 PerCP-Cy5.5 (BD, cat#550954) and anti-CD8α APC-H7 (BD, cat#557654). For detection of cytokines anti-IFN-γ PE (BD, cat#554412), anti-TNF-α PE-Cy7 (557644) and anti-IL-2 APC (BD, cat#554429).

Eukaryotic cell lines

Policy information about cell lines

Cell line source(s)	human embryonic kidney cell (Expi293F and HEK293) and MRC-5 cells

Authentication	the cell line was authenticated according to the CoA of the manufacturer
Mycoplasma contamination	All cell lines tested negative for mycoplasma contamination
Commonly misidentified lines (See ICLAC register)	N.A.

Animals and other organisms

Policy information about studies involving animals; ARRIVE guidelines recommended for reporting animal research

Laboratory animals	Female BALB/c or C57BL6 mice (specific pathogen-free), aged 8-12 weeks at the start of the study were purchased from Charles River laboratories (Sulzfeld, Germany).
Wild animals	The study did not involve laboratory animals
Field-collected samples	The study did not involve samples collected from the field
Ethics oversight	Animal experiments were approved and conducted in accordance with the European guidelines (EU directive on animal testing 86/609/EEC) and local Dutch legislation on animal experiments.

Note that full information on the approval of the study protocol must also be provided in the manuscript.

Flow Cytometry

Plots

Confirm that:

☒ The axis labels state the marker and fluorochrome used (e.g. CD4-FITC).

☒ The axis scales are clearly visible. Include numbers along axes only for bottom left plot of group (a 'group' is an analysis of identical markers).

☒ All plots are contour plots with outliers or pseudocolor plots.

☒ A numerical value for number of cells or percentage (with statistics) is provided.

Methodology

Sample preparation	MRC-5 cells were transfected. Splenocytes were obtained by disaggregation of spleens with the gentleMACS dissociator.
Instrument	FACS Canto instrument (BD Biosciences)
Software	FlowJoTM Software version 10.06.1 (Becton, Dickinson and Company)
Cell population abundance	A minimum of 1,000 cells per subpopulation were collected for including samples in analyses
Gating strategy	Data were plotted as median fluorescence intensity of the MRC-5 single, live cell population

☒ Tick this box to confirm that a figure exemplifying the gating strategy is provided in the Supplementary Information.
